# L-Carnitine Combined with Leucine Supplementation Does Not Improve the Effectiveness of Progressive Resistance Training in Healthy Aged Women

**DOI:** 10.1007/s12603-022-1848-y

**Published:** 2022-10-12

**Authors:** A.K. Sawicka, J. Jaworska, B. Brzeska, A. Sabisz, E. Samborowska, M. Radkiewicz, E. Szurowska, P.J. Winklewski, A. Szarmach, Robert A. Olek

**Affiliations:** 1 Applied Cognitive Neuroscience Lab, Department of Human Physiology, Faculty of Health Sciences, Medical University of Gdansk, Gdansk, Poland; 2 Department of Physiology, Faculty of Medicine, Medical University of Gdansk, Gdansk, Poland; 3 IInd Department of Radiology, Faculty of Health Sciences, Medical University of Gdansk, Gdansk, Poland; 4 Department of Biology and Pharmaceutical Botany, Faculty of Pharmacy, Medical University of Gdansk, Gdansk, Poland; 5 Department of Human Physiology, Faculty of Health Sciences, Medical University of Gdansk, Gdansk, Poland; 6 Mass Spectrometry Laboratory, Institute of Biochemistry and Biophysics, Polish Academy of Sciences, Warsaw, Poland; 7 Department of Athletics, Strength, and Conditioning, Poznan University of Physical Education, Krolowej Jadwigi 27/39, 61-871, Poznan, Poland

**Keywords:** Sarcopenia, trimethylamine-N-oxide, insulin-like growth factor-1, myostatin, decorin, exercise training, muscle cross-sectional area

## Abstract

**Objectives:**

To evaluate the effect of L-carnitine (LC) in combination with leucine supplementation on muscle strength and muscle hypertrophy in aged women participating in a resistance exercise training (RET) program.

**Design/Setting/Participants:**

Thirty-seven out of sixty (38.3% dropout) healthy women aged 60–75 years (mean 67.6 ± 0.7 years) completed the intervention in one of three groups. One of the supplemented groups received 1 g of L-carnitine-L-tartrate in combination with 3 g of L-leucine per day (LC+L group; n = 12), and the second supplemented group received 4 g of L-leucine per day (L group; n = 13). The control group (CON group; n = 12) received no supplementation.

**Intervention:**

All three groups completed the same RET protocol involving exercise sessions twice per week for 24 weeks.

**Measurements:**

Before and after the experiment, participants performed isometric and isokinetic muscle strength testing on the Biodex dynamometer. The cross-sectional areas of the major knee extensors and total thigh muscles were assessed using magnetic resonance imaging. Fasting serum levels of insulin-like growth factor-1 (IGF-1), myostatin and decorin, and plasma levels of total carnitine (TC) and trimethylamine-N-oxide (TMAO) levels were measured.

**Results:**

The 24-week RET significantly increased muscle strength and muscle volume, but the group and time interactions were not significant for the muscle variables analyzed. Plasma total carnitine increased only in the LC+L group (p = 0.009). LC supplementation also caused a significant increase in plasma TMAO, which was higher after the intervention in the LC+L group than in the L (p < 0.001), and CON (p = 0.005) groups. The intervention did not change plasma TMAO concentration in the L (p = 0.959) and CON (p = 0.866) groups. After the intervention serum decorin level was higher than before in both supplemented groups combined (p = 0.012), still not significantly different to post intervention CON (p = 0.231). No changes in serum IGF-1 and myostatin concentrations and no links between the changes in blood markers and muscle function or muscle volume were observed.

**Conclusions:**

LC combined with leucine or leucine alone does not appear to improve the effectiveness of RET.

## Introduction

**A**ge-related changes in anabolic and catabolic processes are associated with progressive loss of muscle mass, strength, and function (1). Exercise training is an intervention that can prevent or even reverse the muscle-wasting process (2), and the greatest effect of exercise on this process in older adults results from resistance exercise training (RET) (3). RET-accelerated skeletal muscle hypertrophy is controlled by hormones and growth factors (4). Protein synthesis is induced by insulin-like growth factor-1 (IGF-1) and inhibited by myostatin. Myostatin activity is suppressed by a small leucine-rich proteoglycan, decorin (5), which is secreted in response to exercise (6).

RET in combination with nutritional intervention provides a better stimulus for maintaining muscle strength and mass than RET alone (7). Leucine is an important regulator of protein synthesis (8). In short-term studies, leucine intake stimulates muscle protein synthesis (9). However, longer studies of older adults, supplemented with leucine for 3 months (10) or 6 months (11) have not confirmed the effects of supplementation on muscle strength or mass. This discrepancy might be attributable to the fact that the other factors, such as growth factors and hormones, satellite cells and neuromuscular factors may be required for the translation of acute increases in protein synthesis into chronic increases in muscle mass (12).

Leucine has also been reported to increase insulin levels (13), and insulin is required for the transport of L-carnitine (LC) into muscles (14). In addition, animal studies have shown that the metabolic pathways involved in muscle protein balance can be upregulated by LC supplementation (15, 16), possibly through its ability to elevate the circulating level of IGF-1, a potential promotor of muscle protein balance (17). A recent study reported increases in muscle mass and strength in older adults consuming LC mixed with leucine, creatine, and vitamin D for 8 weeks (18). Whether similar results can be obtained with LC combined with leucine during the course of an RET protocol remains unknown.

A crucial role in the development of muscle loss during aging may play gut microbiota (19), also involved in the metabolism of orally administered LC to circulating trimethylamine-N-oxide (TMAO) (20). In vitro studies indicate that TMAO can increase protein synthesis (21), or modulate myosin ATPase activity (22, 23). Interestingly, TMAO has been shown to be taken up by human skeletal muscles (24), and prolonged LC supplementation elevates circulating TMAO levels in healthy aged women (25).

The main purpose of our study was to examine the effects of the combination of LC and leucine on muscle volume and strength in healthy aged women undertaking RET twice a week for 24 weeks. We measured circulating IGF-1, myostatin, decorin, and TMAO levels to identify potential confounding factors of muscle cross-sectional area (CSA) and function in aging. We hypothesized that LC combined with leucine supplementation would alter peripheral factors that would be manifested as skeletal muscle changes in older women. We also hypothesized that an increased TMAO level induced by LC treatment would affect the force production over time.

## Materials and Methods

### Ethics approval and consent to participate

The study was conducted in accordance with the Declaration of Helsinki. The protocol was approved by the Independent Bioethics Commission for Research at the Medical University of Gdansk (NKBBN/354-201/2017) and was registered in the ClinicalTrials.gov Registry (NCT03907592). All participants were informed about the procedures, risks, and expected outcomes before starting the experimental procedure and gave their written informed consent for participation. Partial results of this study have been reported previously (26).

### Sample size

The sample size calculation was carried out by G*Power 3. This study was designed to detect a moderate effect size (f = 0.3) for muscle mass, muscle strength, and physical performance. Using the analysis of variance (ANOVA) for repeated measures, within-between interaction, setting the α-error to 0.05, the power to 85% and 3 groups, the minimal sample size was estimated at 36. Considering at least 20% dropout rate (27), a total of 60 participants were recruited for this trial.

### Participants

**Figure 1:**
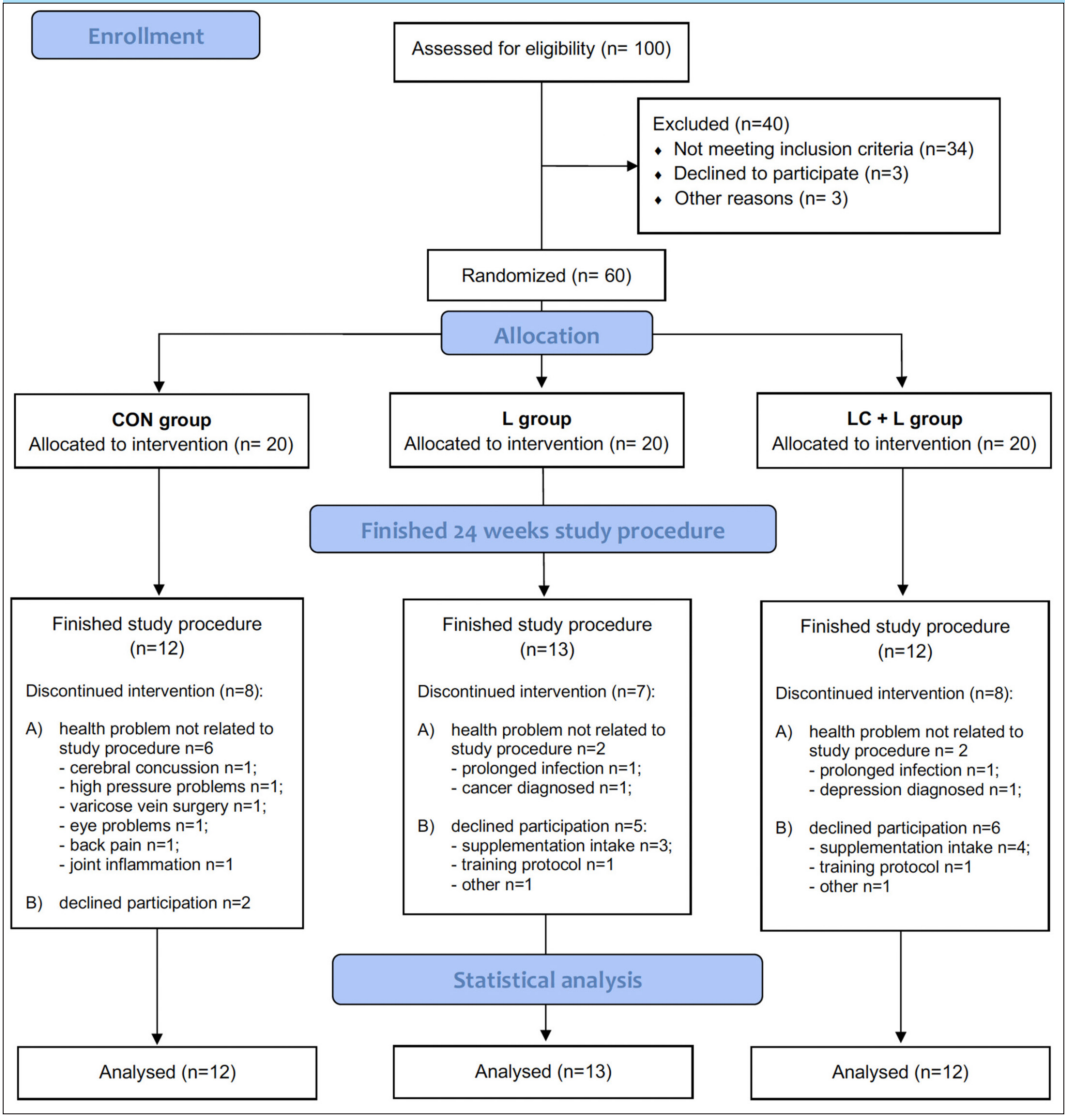
Flow chart of participant recruitment and participation in the study

Participants were recruited through local advertisements between April and June 2017. Volunteers with a chronic disease (such as cardiovascular disease, liver or kidney disease, gastrointestinal disorder, including stomach ulcer or erosions, cancer, diabetes, disease of the musculoskeletal system, and other severe chronic diseases), with metal body implants, or who smoked were excluded. A short questionnaire was used for the assessment of habitual physical activity. Additional activities were converted into metabolic equivalents (METs). Volunteers with low and moderate physical activity and without a professional sports history were included in the study. All included participants presented a physician's certificate indicating a lack of contradictions to strength training. Sixty women aged 60–75 years (mean 67.6 ± 0.7 years) were examined at the beginning of the study protocol (Fig. 1). Height was measured to the nearest 0.1 cm with a portable stadiometer. Weight was measured using a bioelectrical impedance analyzer (InBody720, InBody Co., Ltd., Seoul, Korea). Body mass index was calculated by dividing weight (kg) by height squared (m^2^). The characteristics of the subjects enrolled are shown in Table S1.

### Experimental design and study procedure

After the initial screening, participants were randomly assigned to one of two supplemented groups, LC in combination with leucine (LC+L group; n = 20), or leucine alone (L group; n = 20) or control group (CON group; n = 20). Because of LC poor bioavailability, LC intake should be combined with insulinogenic compound, and the supplementation protocol should take about 100 days to increase muscle carnitine content by ∼ 10% (28). In our study, leucine was used as an insulinogenic compound (13), which could potentially improve LC transport to skeletal muscles. Therefore, the participants were supplemented with either 1 g of L-carnitine-L-tartrate and 3 g of leucine per day (LC+L group) or 4 g of leucine per day (L group) for 24 weeks in a doubleblind fashion. The supplements were encapsulated in identical gelatin capsules, and the supplement packages were coded so that neither the investigators nor the participants were aware of the contents until completion of the analysis. The participants received the packages in separate portions every 2 weeks and were instructed to consume the supplements once a day with their main meal. Adherence to the supplementation protocol was based on information about the unused supplements. In parallel, the CON group participated in the RET but did not receive the supplements.

During the week before starting the training protocol, and the week following the last training session, all participants performed the series of tests described below (Fig. S1). Fasting blood samples were obtained and, after a standardized breakfast, strength tests were performed. A magnetic resonance imaging (MRI) scan was performed on a separate day, but no earlier than 2 days after muscle strength testing.

### RET protocol

The training sessions were held in groups of up to 12 people twice a week in a commercial gym. Each participant attended on Mondays and Wednesdays, or on Tuesdays and Thursdays. Each class was conducted by professional coaches. Over the 24 weeks, each participant had participated in 48 training sessions, each lasting 45–60 min.

The RET protocol was based on a previously described procedure (29). Each training session started with a 10 min warm-up on a treadmill (walking), and participants then performed three sets of four exercises: leg press, leg extension, shoulder press or horizontal row, and chest press or lateral pulldown. The leg press and leg extension were performed at every training session, but the shoulder press and lateral pulldown were performed only on Monday or Tuesday, and horizontal row and chest press only on Wednesday or Thursday. Each session ended with a 10 min cooldown on a cycle ergometer.

A one-repetition maximum test (1RM) was performed, according to National Strength and Conditioning Association guidelines (30), before and then every 6 weeks of the training protocol. In total each participant performed 1RM five times. During the first 2 weeks, the workload was set at 65% of 1RM for each exercise, and the exercise was performed in three sets of 10–12 repetitions. After 2 weeks, the workload was increased to 80% of 1RM, and each exercise was performed in three sets of 6–8 repetitions. All three groups completed the same RET protocol.

### Skeletal muscle strength

All strength tests were performed on the Biodex System 4 Pro dynamometer (Biodex Medical Systems, Inc., Shirley, NY, USA). Before the start of each testing session, the Biodex was calibrated according to the manufacturer's specifications. Before testing, the participant performed a 5 min warm-up at 50 W on a mechanically braked cycle ergometer (Monark, Vansbro, Sweden). The test started with an isometric test at a 90° knee angle, followed by an isokinetic test at 60°/s for the dominant leg. Participants were stabilized with two shoulder straps, a waist strap, and a thigh strap. The rotational axis of the knee was aligned with the center of the dynamometer shaft. Adjustments were made to the length of the knee attachment to ensure that the ankle strap was proximal to the lateral and medial malleoli and comfortable for the participant. Gravity correction was used for all trials. Verbal encouragement was provided during all tests (31). Peak torque was measured by performing maximum voluntary contractions (MVC) during isometric knee extension. The test comprised a maximum 4 s knee extensor isometric contraction, which was repeated three times separated by a 20 s recovery. To assess muscle isokinetic strength, the participant completed five repetitions at a speed of 60°/s (32). During concentric isokinetic leg extension, the total work of the five repetitions was recorded (33).

### Cross-sectional area

The dominant leg was analyzed using a 1.5 T Siemens MAGNETOM Aera MRI scanner (Siemens, Munich, Germany) with the body 18 and spine 32 coils and Auto Coil Select mode on. The study protocol included, the following sequences: T1-weighted turbo spin echo coronal (voxel 1.8 × 1.8 × 4 mm, FOV 250 × 200 mm, TE 17 ms, TR 500 ms, NSA 2), T2 space transverse (voxel 0.8 × 0.8 × 3 mm, TE 96 ms, TR 1600 ms, NSA 1.4) and T1 VIBE Dixon (voxel 0.7 × 0.7 × 2.5 mm, TE 2.39 and 4.77 ms, TR 6.89 ms, NSA 1). The examination covered the area from the knee joint level to the end of the hip joint, and all sequences were performed twice and then combined into one composite image. The Dixon sequence comprised in-phase and out-of-phase images and reconstructed water-only and fat-only images.

After all data collection, the 2/3 upper femur height, as specified for measuring the maximal strength of the knee extensors (34), was determined by an experienced radiologist. Subsequently, the CSAs of the total thigh muscle (CSA TM) and of vastus lateralis, vastus intermedius, and vastus medialis, as the main knee extensors (CSA KE), were measured using an OsiriX Life (Pixmeo SARL, Bernex, Switzerland). All evaluations were performed by two independent investigators who were blinded to the intervention. The interindividual coefficient of variation of the analysis was 1.3%.

### Blood collection and analysis

Fasting blood samples were taken from the antecubital vein into BD Vacutainer® tubes (Becton, Dickinson and Company, Franklin Lakes, NJ, USA). After collection, the samples were centrifuged at 2000 g at 4°C for 10 min, and aliquots were stored at -80°C for later analyses. Plasma TMAO concentration was measured as described previously (35). For total carnitine (TC), 5 µL of the sample (plasma, calibration points) was transferred into a 1.5 mL test tube, then 200 µL of acetonitrile containing the internal standard was added for protein precipitation, and 100 µL of 1 M KOH in methanol was added to hydrolyze acylcarnitines. The solution was incubated at 50°C for 60 min, and 100 µl of 1 M HCl in methanol was added to neutralize the mixture (36). Samples were centrifuged for 2 min at 14000 rpm and injected into liquid chromatography-mass spectrometry (LC-MS/MS) system in the Mass Spectrometry Laboratory, Institute of Biochemistry and Biophysics, Polish Academy of Sciences (Warsaw, Poland). Serum IGF-1, myostatin, and decorin concentrations were measured using commercially available enzyme immunoassay kits (total IGF-1, cat. #DG100, myostatin, cat. #DGDF80, decorin cat. #DY143 and #DY008; R&D Systems, Minneapolis, MN, USA).

### Diet

Three-day food records were self-reported for two weekdays and one weekend day at the beginning of the study. Participants were instructed to note the amounts of food and beverages consumed. The diet was analyzed in terms of the amount of energy, protein, carbohydrates (CHO), and fat consumed.

### Statistical analysis

Participants included in the statistical analyses completed a minimum of 80% of the training sessions. All calculations were performed using Statistica 13.1 software (Dell Inc., Tulsa, OK, USA). The normality of the data distribution was established using the Shapiro-Wilk test. Repeated-measures analysis of variance (ANOVA) was used for normally distributed data, and the Friedman repeated-measures ANOVA by ranks was performed for nonnormally distributed data. The Kruskal-Wallis ANOVA was used to compare groups at the same time point. Correlations between the changes in absolute values from before to after the RET intervention were calculated using Pearson and Spearman correlation tests for normally and nonnormally distributed data, respectively. A probability level of p < 0.05 was considered to be significant. All data are expressed as mean ± SD, unless otherwise stated.

## Results

The study protocol was completed by 37 participants (Fig. 1). Despite the high dropout (38.3%), the characteristics of the analyzed subjects did not differ between the groups (Table 1). The direct comparison between subjects who completed the study protocol and those who dropout the study also indicated no differences (Table S2).

**Table 1:** Baseline characteristics and dietary composition of the participants

	**CON (n = 12)**	**L(n = 13)**	**LC+L (n = 12)**	**p**
Age (years)	65.8 ± 2.6	67.9 ± 2.1	68.0 ± 2.7	0.067
Weight (kg)	70.6 ± 11.5	68.9 ± 13.2	73.0 ± 14.2	0.743
Height (cm)	162.8 ± 6.3	158.9 ± 4.6	159.5 ± 5.5	0.176
BMI	26.6 ± 3.9	27.2 ± 4.7	28.7 ± 5.7	0.551
METs	2.6 ± 1.5	3.1 ± 1.1	3.3 ± 1.6	0.769
Diet				
Energy (MJ/d)	6.5 ± 2.0	6.6 ± 0.9	7.0 ± 1.8	0.742
CHO (g/kg/d)	2.6 ± 1.3	2.9 ± 1.0	2.7 ± 1.2	0.743
Protein (g/kg/d)	1.0 ± 0.3	1.1 ± 0.3	1.1 ± 0.3	0.852
Fat (g/kg/d)	0.9 ± 0.4	0.9 ± 0.3	1.0 ± 0.5	0.821


CON: control group; L: leucine supplemented group; LC+L: L-carnitine and leucine supplemented group; BMI: Body mass index; METs: Metabolic equivalents of additional daily physical activity; CHO: carbohydrates

### Effect of the RET intervention on muscle strength and CSA

RET caused significant increases in isometric peak torque (p = 0.009), isokinetic peak torque (p < 0.001), average power (p < 0.001), total work (p < 0.001) (Table 2). RET also had a significant effect on muscle hypertrophy, as measured by CSA TM (p = 0.005) and CSA KE (p = 0.006). However, the group and time interactions were not significant for the muscle variables analyzed (Table 2).

**Table 2:** Measurements of isometric and isokinetic strength and cross-sectional area in the dominant leg before and after the RET protocol

	**CON (n = 12)**			**L (n = 13)**			**LC+L (n = 12)**			**p group × time**
	**pre**	**post**	**% change**	**pre**	**post**	**% change**	**pre**	**post**	**% change**	
Isometric										
Peak Torque (Nm)	153 ± 31	159 ± 39	4.0 ± 17.8	144 ± 34	157 ± 51	7.9 ± 13.1	154 ± 30	170 ± 39	11.4 ± 24.3	0.663
Isokinetic										
Peak Torque (Nm)	127 ± 18	134 ± 30	5.2 ± 17.3	117 ± 29	127 ± 34	8.8 ± 9.5	124 ± 19	135 ± 20	9.4 ± 12.4	0.814
Average power (W)	53 ± 11	56 ± 12	8.5 ± 27.8	48 ± 10	57 ± 16	19.5 ± 19.2	51 ± 11	61 ± 10	19.8 ± 15.9	0.209
Total work (J)	483 ± 91	529±116	10.9 ± 26.0	478 ± 87	512±135	6.8 ± 16.4	484±106	557 ± 87	17.6 ± 19.9	0.509
Cross-sectional area										
Thight muscles (cm^2^)	114 ± 15	116 ± 16	2.4 ± 4.8	112 ± 18	114 ± 18	2.0 ± 3.7	113 ± 13	116 ± 13	2.5 ± 5.3	0.966
Vastus muscles (cm^2^)	38.3 ± 4.7	39.3 ± 5.2	2.9 ± 8.7	36.9 ± 8.3	37.7 ± 7.9	2.5 ± 5.1	36.6 ± 5.7	38.5 ± 5.8	5.6 ± 7.0	0.567


CON: control group; L: leucine supplemented group; LC+L: L-carnitine and leucine supplemented group;- % change: percent changes between pre- and post- intervention values

### Changes in blood markers

**Figure 2:**
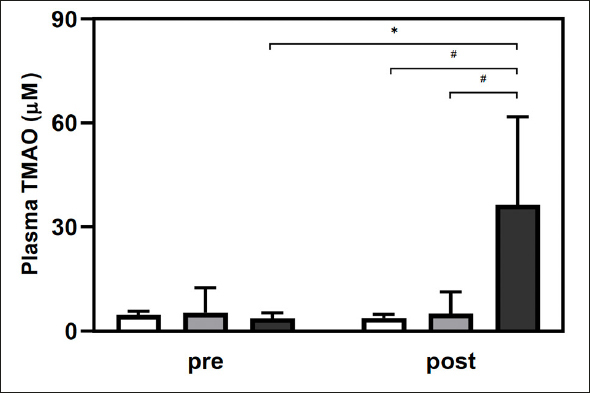
Plasma trimethylamine-N-oxide (TMAO) presented as means (± SD) in CON (white bars), L (gray bars), and LC+L (black bars) groups before and after 24 weeks of the RET *p < 0.001, compared with before the intervention in the same group; #p < 0.005, compared between groups at the same time.

TMAO level increased only in the LC+L group (p < 0.001) and was higher after the intervention in the LC+L group than in the L (p < 0.001), and CON (p = 0.005) groups. The intervention did not change plasma TMAO concentration in the L (p = 0.959) and CON (p = 0.866) groups (Fig. 2). LC supplementation also caused a significant increase in plasma TC (CON 2.3 ± 6.6, L 0.4 ± 9.1, LC+L 9.9 ± 6.3 µmol/L; p = 0.009). No differences were observed in circulating IGF-1 (group p = 0.790, time p = 0.190, group*time p = 0.757), myostatin (group p = 0.255, time p = 0.508, group*time p = 0.619), and decorin (group p = 0.380, time p = 0.030, group*time p = 0.075) levels (Table 3). After the intervention serum decorin level was higher than before in both supplemented groups combined (p = 0.012), still not significantly different to post intervention CON (p = 0.231). Other parameters did not change significantly (Table S3).

**Table 3:** Serum biomarkers before and after the RET protocol, with calculated percent changes between pre- and post- intervention values

	**CON (n = 12)**			**L (n = 13)**			**LC+L (n = 12)**			**p group × time**
	**pre**	**post**	**% change**	**pre**	**post**	**% change**	**pre**	**post**	**% change**	
IGF-1 (µg/L)	92 ± 28	93 ± 29	1.6 ± 17.8	83 ± 22	88 ± 28	6.8 ± 19.9	87 ± 27	92 ± 25	11.3 ± 36.3	0.757
Myostatin (µg/L)	3.2 ± 1.2	3.3 ± 1.2	5.1 ± 24.4	4.3 ± 1.8	4.2 ± 1.6	2.2 ± 24.4	3.4 ± 1.6	3.7 ± 1.6	12.2 ± 27.5	0.619
Decorin (µg/L)	8.1 ± 1.3	7.9 ± 1.9	−2.5 ± 15.5	8.4 ± 1.4	9.2 ± 1.8	9.4 ± 11.1	8.0 ± 1.6	8.7 ± 1.3	9.9 ± 16.9	0.075


CON: control group; L: leucine supplemented group; LC+L: L-carnitine and leucine supplemented group; % change: percent changes between pre- and post- intervention values

### Correlations between skeletal muscle parameters and circulating markers

The elevation in plasma TC level positively correlated with the change in plasma TMAO level (rho = 0.595, p < 0.001; Table S4). However, no other significant correlations between the changes in circulating markers and muscle function or muscle volume were noted (Table S4). In addition, the change in muscle volume from before to after the RET did not correlate with improvements in strength test variables (Table S4).

## Discussion

The main findings of this study were that LC combined with leucine or leucine alone does not improve the effectiveness of RET; the increase in circulating TC level was associated with the increase in TMAO level; the changes in circulating markers did not correlate with muscle function or volume; supplemented groups analyzed together had higher decorin levels.

LC supplementation has been proposed as a potential promotor of muscle protein balance (37). The suggested mechanism involves activation of the mammalian target of rapamycin kinase (mTOR) pathway in skeletal muscle (for review see (38)). Activation of the mTOR signaling pathway, which is associated with increases in muscle mass and strength, was observed after 8 weeks of supplementation with LC in combination with leucine, creatine, and vitamin D without additional physical activity (18). Leucine is an important regulator of skeletal muscle anabolism (8, 39). However, long-term studies of older adults supplemented with leucine for 6 months (11), even when combined with strength training twice a week (40), have not produced positive effects on muscle mass or strength. Similarly, we observed no additional increase in muscle volume or function in the group supplemented with leucine alone (L group) or with leucine in combination with LC (LC+L group) after the 24 weeks of RET. Considering these observations, it cannot be ruled out that previous effects (18) may have been related to supplementation with creatine, which delays muscle atrophy and improves strength during aging (41, 42), or to vitamin D, which can improve muscle strength in people aged ≥65 years (43). Especially that supplementation with a mixture containing creatine and vitamin D, among others, for 12 weeks without additional physical activity improved muscle strength and muscle power in healthy elderly humans (44).

Circulating IGF-1 has been suggested as a potential mechanism underlying the beneficial effects of LC supplementation on skeletal muscle protein turnover (37, 38). An elevation in circulating IGF-1 level after LC supplementation was observed in animal (15, 17) and clinical (45) studies. LC supplementation for 10 weeks in prefrail older people (46), and for 24 weeks in healthy older women (47) did not affect circulating IGF-1 levels. Despite improvements of strength and skeletal muscle hypertrophy following RET (3) evidence to support a role of circulating IGF-1 is inconsistent. Increased IGF-1 level was reported in older people following 12 months (48) and 8 months (49) of RET. By contrast, no change in serum IGF-1 was found in older people after 6 months of RET (50, 51), even in those supplemented with 20.7 g of protein (3 g leucine, >10 g of essential amino acids), 9.3 g of CHO, 3 g of fat, and vitamins and minerals (51). Similarly, in our study, RET alone and RET with supplementation did not change blood IGF-1 levels. In addition, we observed no effect of LC on thigh muscle volume following 24 weeks of RET combined with leucine supplementation. Correspondingly, a higher LC dose combined with an insulinogenic beverage (44.4 g CHO, 13.8 g protein) and moderate-intensity cycling over 25 weeks did not affect lean body mass in older men (52). By contrast, 2 g of LC per day for 6 months increased fat-free mass in centenarians (53). The contradictory results may be related to the age of the participants. Accelerated skeletal muscle mass loss is observed in humans aged ≥80 years, and is strongly associated with a decrease in serum IGF-1 level (54). Therefore, LC supplementation does not appear to affect fasting serum IGF-1 level in people aged <80 years (46, 47) but may be effective in centenarians. Importantly, in longitudinal studies, older people may not ingest sufficient protein (55) or energy (56). Although energy intake and dietary composition did not differ between groups in our study, a protein intake ∼1.0g/kg/ day may limit the extent of adaptation to RET (57).

Myostatin is a negative regulator of skeletal muscle growth (58) and may be downregulated by decorin (5). Increased muscle decorin expression correlates with improvement in leg press performance (6). Acute resistance exercise elevates plasma decorin at the end of the session, although 120 min after the end of exercise it returns to baseline level (6) and resting plasma decorin level is not affected by 5 weeks exercise intervention (59). Similarly, in our study, decorin level did not change after 24 weeks of RET in the CON group but increased significantly in both supplemented groups. However, we found no associations between the differences in circulating decorin levels and changes in muscle volume and function, and no effects of RET or supplementation on myostatin levels. The lack of association between exercise performance and circulating mediators may indicate that exercise increases muscle strength by predominately locally derived mediators rather than circulating factors (60).

The recent meta-analysis of 37 randomized controlled trails indicated that LC supplementation might affect body weight and composition, with a dose of 2 g LC per day providing the maximum effect in adults (61). In fact, LC supplementation (2g/day) in combination with a resistance training program (4 days/week) applied to healthy men (age range 18–40 years), for 9 weeks caused statistically significant improvements in bench and leg presses. There were no differences between the supplemented and placebo groups, but the number of repetitions and lifting volume increased in the LC group compared to baseline values (62). In the present study, the applied daily LC dose was lower than previously reporting enhancement in muscle strength of healthy humans (18, 62), but the total amount of LC consumed was higher due to prolonged period of supplementation. Importantly, LC needs insulin for transportation into muscle (14). Muscle carnitine level increases only when LC is coingested with a large amount of CHO to induce an insulin response (63), and the supplementation protocol should take minimum 100 days (28). Leucine has been reported to increase insulin level (13), and we assumed that 24 weeks of LC supplementation in combination with leucine could improve LC transport into muscle. The increase in insulin levels in our study may have been low compared with a previous study on carnitine supplementation with CHO to induce release of insulin (63). Indeed, the same supplementation protocol did not change skeletal muscle TC level in young participants (unpublished), suggesting that muscle TC was not affected also in the present study.

Prolonged LC treatment elevates fasting plasma TMAO level (25), and TMAO can modulate myosin ATPase activity (22, 23). We hypothesized that LC supplementation would affect muscle strength by increasing circulating TMAO level and modifying ATPase activity. Erickson et al. (64) recently reported a correlation between fasting TMAO level and aerobic capacity in older sedentary adults with obesity. However, we did not observe any significant associations between plasma TMAO level and muscle function parameters. Erickson and colleagues (64) also reported that 12 weeks of exercise training combined with a hypocaloric diet induced a percentage reduction (but not in the absolute level) in fasting TMAO concentration. The similar fasting TMAO levels in the CON and L groups after 24 weeks of RET in the present study suggest a minimal role of exercise in the reduction in plasma TMAO concentration. Nutritional intervention seems to be more important factor for modifying plasma TMAO level. The correlation observed between the increases in plasma TC and TMAO concentrations may be because LC is a substrate for TMAO production (20).

In light of current findings, it should be noted that LC combined with leucine or leucine alone supplementation does not improve the effectiveness of RET in healthy aged women, at least in studied doses. In addition, LC supplementation elevates plasma TMAO level. Studies have shown that TMAO is a risk factor for the development of noncommunicable diseases, but whether it affects the health status of people without any chronic diseases is widely discussed (Coutinho-Wolino et al., 2021, Wang et al., 2021, Loo et al., 2022), and needs further research.

Our study has several limitations. First, we did not evaluate the markers in skeletal muscle. Second, the high dropout and small sample size may have limited the statistical power and the ability to identify differences between groups. Third, CON was not provided a placebo supplement, and was involved only in the training protocol, which made this study partially blinded. Finally, self-reported data were used to monitor dietary intake only once. Given the increase in physical activity levels by the participants, the energy intake may have also increased, which could have resulted in changes in the dietary macronutrient composition.

## Conclusions

LC combined with leucine or leucine alone supplementation does not improve the efficacy of RET in healthy aged women. Leucine supplementation elevated circulating decorin level, but this increase was not associated with serum myostatin level. LC supplementation increased plasma TMAO level. There are no links between changes in circulating markers and muscle function or volume. Further research using combined nutritional and exercise interventions, as well as the creation of targeted recommendations for elderly populations, would be important for personalized medicine.

## Copyright comment

corrected publication 2023

## Electronic Supplementary Material

Supplementary material, approximately 44 KB.
