# Erratum to: L-Carnitine Combined with Leucine Supplementation Does Not Improve the Effectiveness of Progressive Resistance Training in Healthy Aged Women

**DOI:** 10.1007/s12603-022-1878-1

**Published:** 2023-03-01

**Authors:** A.K. Sawicka, J. Jaworska, B. Brzeska, A. Sabisz, E. Samborowska, M. Radkiewicz, E. Szurowska, P.J. Winklewski, A. Szarmach, Robert A. Olek

**Affiliations:** 1 Applied Cognitive Neuroscience Lab, Department of Human Physiology, Faculty of Health Sciences, Medical University of Gdansk, Gdansk, Poland; 2 Department of Physiology, Faculty of Medicine, Medical University of Gdansk, Gdansk, Poland; 3 IInd Department of Radiology, Faculty of Health Sciences, Medical University of Gdansk, Gdansk, Poland; 4 Department of Biology and Pharmaceutical Botany, Faculty of Pharmacy, Medical University of Gdansk, Gdansk, Poland; 5 Department of Human Physiology, Faculty of Health Sciences, Medical University of Gdansk, Gdansk, Poland; 6 Mass Spectrometry Laboratory, Institute of Biochemistry and Biophysics, Polish Academy of Sciences, Warsaw, Poland; 7 Department of Athletics, Strength, and Conditioning, Poznan University of Physical Education, Krolowej Jadwigi 27/39, 61-871, Poznan, Poland

The online version of the original article can be found at https://doi.org/10.1007/s12603-022-1848-y

The original online version of this article was revised: Mistakenly given catalog no. DY788-05 has been changed for DGDF80.

