# Supplementary material for: L-Carnitine Combined with Leucine Supplementation Does Not Improve the Effectiveness of Progressive Resistance Training in Healthy Aged Women
Source: J Nutr Health Aging. 2022 Oct 12;26(10):945–53. doi: 10.1007/s12603-022-1848-y (PMC12275549; doi:10.1007/s12603-022-1848-y)
Supplement: Supplementary file 1 — Supplementary material, approximately 44 KB. [file mmc1.docx]

**Supplementary material**

Table S1. Baseline characteristics of the enrolled participants. Values presented as mean ± SD. One-way ANOVA for normally distributed data, and Kruskal-Wallis ANOVA for nonnormally distributed data were used.

|  | CON (n = 20) | L (n = 20) | LC+L (n = 20) | p |
| --- | --- | --- | --- | --- |
| Age (years) | 66.8 ± 3.3 | 67.8 ± 2.3 | 67.8 ± 2.7 | 0.425 |
| Weight (kg) | 71.2 ± 10.7 | 69.2 ± 12.4 | 70.8 ± 14.3 | 0.870 |
| Height (cm) | 161.7 ± 5.6 | 158.5 ± 4.0 | 159.0 ± 5.8 | 0.123 |
| BMI | 27.2 ± 3.7 | 27.5 ± 4.4 | 27.9 ± 5.2 | 0.878 |
| METs ^N^ | 2.7 ± 1.4 | 3.0 ± 1.4 | 3.2 ± 1.4 | 0.638 |

CON: control group; L: leucine supplemented group; LC+L: L-carnitine and leucine supplemented group; BMI: Body mass index; METs: Metabolic equivalents of additional daily physical activity; N: nonnormally distributed data


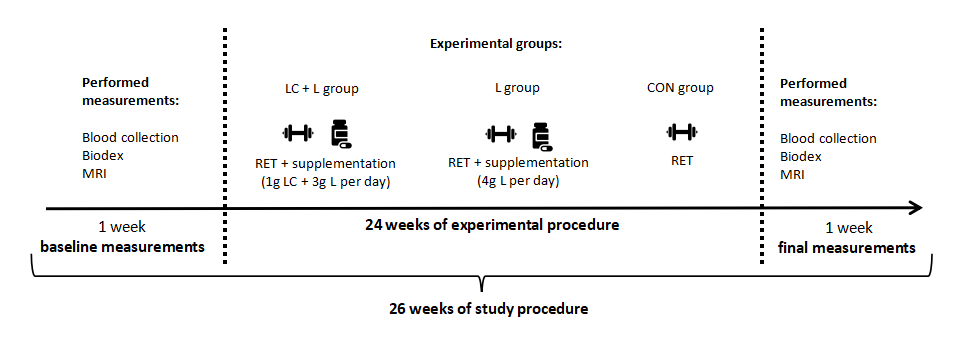


Figure S1. Timeline of the experimental protocol. MRI: magnetic resonance imaging; RET: resistance training; LC: L-carnitine; L: leucine.

Table S2. Comparison of baseline values of participants included and dropped out of the study in each group. Values presented as mean ± SD.

|  | CON analyzed  (n = 12) | CON dropout  (n = 8) | p | L  analyzed  (n = 13) | L  dropout  (n = 7) | p | LC+L analyzed  (n = 12) | LC+L dropout (n = 8) | p |
| --- | --- | --- | --- | --- | --- | --- | --- | --- | --- |
| Age (years) | 65.8  ± 2.6 | 68.1  ± 3.8 | 0.128 | 67.9  ± 2.1 | 67.4  ± 2.7 | 0.658 | 68.0  ± 2.7 | 67.4  ± 2.7 | 0.619 |
| Weight (kg) | 70.6  ± 11.5 | 72.0  ± 10.0 | 0.774 | 68.9 ±13.2 | 69.7  ± 11.8 | 0.899 | 73.0  ± 14.2 | 67.6  ± 14.7 | 0.426 |
| Height (cm) | 162.8  ± 6.3 | 159.9  ± 4.2 | 0.259 | 158.9  ± 4.6 | 157.6  ± 2.7 | 0.485 | 159.5  ± 5.5 | 158.3  ± 6.5 | 0.649 |
| BMI | 26.6  ± 3.9 | 28.2  ± 3.4 | 0.369 | 27.2  ± 4.7 | 28.0  ± 4.1 | 0.724 | 28.7  ± 5.7 | 26.8  ± 4.3 | 0.434 |
| METs | 2.6  ± 1.5 | 2.8  ± 1.3 | 0.829 | 3.1  ± 1.1 | 2.8  ± 1.9 | 0.724 | 3.3  ± 1.6 | 3.2  ± 1.3 | 0.879 |

CON: control group; L: leucine supplemented group; LC+L: L-carnitine and leucine supplemented group; BMI: Body mass index; METs: Metabolic equivalents of additional daily physical activity

Table S3. Comparison of results between the CON and supplemented groups.

|  | CON (n = 12) | | | L & LC+L (n = 25) | | | *p*  group x time |
| --- | --- | --- | --- | --- | --- | --- | --- |
|  | pre | post | % change | pre | post | % change |  |
| Isometric | | | | | | | |
| Peak Torque (Nm) | 153 ± 31 | 159 ± 39 | 4.0 ± 17.8 | 149 ± 32 | 163 ± 46 | 9.6 ± 19.0 | 0.384 |
| Isokinetic | | | | | | | |
| Peak Torque (Nm) | 127 ± 18 | 134 ± 30 | 5.2 ± 17.3 | 120 ± 25 | 131 ± 28 | 9.1 ± 10.7 | 0.525 |
| Average power (W) | 53 ± 11 | 56 ± 12 | 8.5 ± 27.8 | 49 ± 10 | 59 ± 13 | 19.6 ±17.3 | 0.075 |
| Total work  (J) | 483 ± 91 | 529 ± 116 | 10.9 ±26.0 | 481 ± 95 | 534 ± 115 | 12.0 ±18.6 | 0.817 |
| Cross-sectional area | | | | | | | |
| Tight muscle  (cm^2^) | 114 ± 15 | 116 ± 16 | 2.4 ± 4.8 | 112 ± 16 | 115 ± 16 | 2.3 ± 4.4 | 0.881 |
| Vastus muscles (cm^2^) | 38.3 ± 4.7 | 39.3 ± 5.2 | 2.9 ± 8.7 | 36.8 ± 7.0 | 38.1 ± 6.8 | 4.0 ± 6.1 | 0.759 |
| Serum | | | | | | | |
| IGF-1  (μg/L) | 92 ± 28 | 93 ± 29 | 1.6 ± 17.8 | 85 ± 24 | 90 ± 26 | 8.9 ± 28.4 | 0.452 |
| Myostatin  (μg/L) | 3.2 ± 1.2 | 3.3 ± 1.2 | 5.1 ± 24.4 | 3.8 ± 1.7 | 3.9 ± 1.6 | 7.0 ± 25.9 | 0.990 |
| Decorin  (μg/L) | 8.1 ± 1.3 | 7.9 ± 1.9 | -2.5 ± 15.5 | 8.2 ± 1.5 | 8.9 ± 1.5 ^x^ | 9.6 ± 13.9 | **0.023** |

^x^ p=0.012 comparing to pre-supplementation value

Table S4. Pearson and Spearman (underlined) correlations for the changes from before to after RET.

|  | CSA KE | MVC PT | PT | AP | TW | TMAO | TC | IGF-1 |
| --- | --- | --- | --- | --- | --- | --- | --- | --- |
| CSA TM | 0.790 *** | 0.157 | 0.226 | 0.226 | 0.189 | -0.162 | -0.048 | -0.129 |
| CSA KE | x | 0.239 | 0.236 | 0.226 | 0.141 | 0.046 | 0.035 | -0.318 |
| MVC PT |  | x | 0.558 *** | 0.342 * | 0.424 ** | -0.050 | -0.216 | -0.171 |
| PT |  |  | x | 0.767 *** | 0.744 *** | -0.233 | -0.184 | -0.265 |
| AP |  |  |  | x | 0.744 *** | 0.066 | -0.130 | -0.108 |
| TW |  |  |  |  | x | -0.004 | -0.077 | -0.029 |
| TMAO |  |  |  |  |  | x | 0.595 *** | -0.013 |
| TC |  |  |  |  |  |  | x | 0.118 |

Significant values: * <0.05; ** <0.01; *** <0.001

CSA TM: cross-sectional area of the total thigh muscles; CSA KE: cross-sectional area of the knee extensors; MVC PT: peak torque of maximum voluntary contractions; PT: peak torque; AP: average power; TW: total work; TMAO: trimethylamine-N-oxide; TC: total carnitine; IGF-1: insulin-like growth factor-1;
